# Comprehensive Analysis of SIGLEC-15 and PD-L1 Expression Identifies Distinct Prognostic Profiles in Gastric Cancer

**DOI:** 10.3390/ijms26178637

**Published:** 2025-09-05

**Authors:** Andreea-Raluca Cozac-Szőke, Andreea Cătălina Tinca, Anca Negovan, Alexandra Vilaia, Dan-Alexandru Cozac, Iuliu-Gabriel Cocuz, Adrian Horațiu Sabău, Raluca-Diana Hagău, Diana-Maria Chiorean, Andreea-Bianca Lazar, Sabin Turdean, Emőke-Andrea Szász, Alexandru Nicușor Tomuț, Ovidiu Simion Cotoi

**Affiliations:** 1 Doctoral School of Medicine and Pharmacy, George Emil Palade University of Medicine, Pharmacy, Science, and Technology of Targu Mures, 540142 Targu Mures, Romania; andreea-raluca.szoke@umfst.ro (A.-R.C.-S.); dan-alexandru.cozac@umfst.ro (D.-A.C.); adrian-horatiu.sabau@umfst.ro (A.H.S.); diana.chiorean@umfst.ro (D.-M.C.); 2 Pathophysiology Department, George Emil Palade University of Medicine, Pharmacy, Science, and Technology of Targu Mures, 540142 Targu Mures, Romania; iuliu.cocuz@umfst.ro (I.-G.C.); ovidiu.cotoi@umfst.ro (O.S.C.); 3 Pathology Department, Mures Clinical County Hospital, 540011 Targu Mures, Romania; ralucahagau1995@gmail.com (R.-D.H.); ohii.bianca@yahoo.com (A.-B.L.); 4 Department of Clinical Science-Internal Medicine, George Emil Palade University of Medicine, Pharmacy, Science, and Technology of Targu Mures, 540142 Targu Mures, Romania; ancanegovan@yahoo.com; 5 Department of Infectious Diseases I, Doctoral School, Carol Davila University of Medicine and Pharmacy, 050474 Bucharest, Romania; alexandra.vilaia@gmail.com; 6 Department of Pathology, George Emil Palade University of Medicine, Pharmacy, Science, and Technology of Targu Mures, 540142 Targu Mures, Romania; sabin.turdean@umfst.ro; 7 Department of Histology, George Emil Palade University of Medicine, Pharmacy, Science, and Technology of Targu Mures, 540142 Targu Mures, Romania; emoke.szasz@umfst.ro; 8 Faculty of Medicine, George Emil Palade University of Medicine, Pharmacy, Science, and Technology of Targu Mures, 540142 Targu Mures, Romania; nicusortomut19@gmail.com

**Keywords:** gastric cancer, SIGLEC-15, PD-L1, immunohistochemistry, immune checkpoint, immunotherapy resistance, tumor microenvironment, prognostic biomarkers

## Abstract

Gastric cancer remains a major global health burden, with limited response rates to current immunotherapies targeting the programmed death-ligand 1 (PD-1/PD-L1) axis. Recent studies have identified sialic acid-binding immunoglobulin-like lectin 15 (SIGLEC-15) as a novel immune checkpoint molecule that may drive immune evasion through PD-L1–independent pathways. This study aimed to evaluate the expression patterns of SIGLEC-15 and PD-L1 in gastric adenocarcinoma and to investigate their associations with clinicopathological features and patient outcomes. We retrospectively analyzed 133 consecutive cases of gastric adenocarcinoma with complete clinicopathologic and follow-up data. Immunohistochemical staining was performed on formalin-fixed tumor samples; SIGLEC-15 expression on tumor cells was quantified by H-score (high expression defined as ≥110) and PD-L1 status by combined positive score (CPS, positive if ≥1). High SIGLEC-15 expression correlated with multiple adverse pathological features, including lymphatic (*p* = 0.003), venous (*p* = 0.030), and perineural invasion (*p* = 0.010), and was associated with significantly poorer 3-year overall survival (hazard ratio = 3.36, *p* < 0.001). While SIGLEC-15 and PD-L1 expression were not mutually exclusive, an inverse relationship was generally observed. Patients with dual positivity (SIGLEC-15 high/PD-L1 CPS ≥ 1) showed the lowest 36-month survival (32%), compared to 56% in the dual-negative group (SIGLEC-15 low/PD-L1 CPS < 1). These results highlight the clinical relevance of SIGLEC-15 as an independent marker of tumor aggressiveness and poor prognosis in gastric adenocarcinoma. Moreover, stratification based on combined SIGLEC-15 and PD-L1 CPS expression revealed that patients co-expressing high levels of both markers experienced the poorest survival outcomes. These findings suggest that the dual assessment of SIGLEC-15 and PD-L1 may enhance prognostic accuracy and support immunotherapeutic decision-making in gastric cancer.

## 1. Introduction

Gastric cancer (GC) remains one of the leading causes of cancer-related mortality worldwide, with significant geographic variation in incidence and outcomes. Despite advances in systemic therapies and surgical techniques, the prognosis for advanced stages remains poor, emphasizing the urgent need for effective biomarkers and personalized treatment strategies [1,2].

Recent data indicate that rising GC levels, even in younger adults, coincides with lifestyle shifts and microbiome dysbiosis. High-salt and nitrite-rich processed diets, tobacco/alcohol use, excess adiposity, and iatrogenic factors (antibiotics, proton pump inhibitors, hypochlorhydria) reshape the gastric microbiota, promoting nitrosative stress, low-grade inflammation, and immune checkpoint activation [3,4]. In this context, the understanding of immune checkpoint molecules is crucial for advancing cancer immunotherapy (IO). Among these markers, SIGLEC-15 (Sialic acid-binding immunoglobulin-like lectin 15) and programmed death-ligand 1 (PD-L1) have gained significant attention due to their immunosuppressive roles [5,6]. Research has demonstrated that PD-L1 expression is frequently associated with unfavorable prognostic outcomes due to its role in diminishing T cell responses, which is a pivotal element of anti-tumor immunity [7,8]. However, the effectiveness of PD-L1 blockade therapies is often limited, with many patients exhibiting primary or acquired resistance to this treatment strategy [9].

Recent insights have highlighted the expression of SIGLEC-15 as a critical factor in the immune microenvironment of tumors. Elevated levels of SIGLEC-15 correlate with poor prognosis in various malignancies, including lung adenocarcinoma, pancreatic cancer, and colorectal cancer, suggesting its involvement in tumor progression and immunosuppression [10,11]. Interestingly, studies have shown that the expression of SIGLEC-15 and PD-L1 can be mutually exclusive in certain tumors, including lung adenocarcinoma and bladder cancer, indicating that these markers might serve different immunosuppressive mechanisms [11,12]. Specifically, in the context of GC, preliminary findings suggest that the expression of SIGLEC-15 may follow a pattern similar to that observed in other malignancies, potentially serving as an alternative pathway for immune escape when PD-L1 is not expressed [13].

Understanding the interplay between SIGLEC-15 and PD-L1 in GC is crucial, given the dual challenges of therapeutic resistance and tumor heterogeneity. Clinically, assessing both markers may provide valuable prognostic information and inform treatment decisions. A lower PD-L1 expression might indicate a reliance on alternate pathways, such as Siglec-15 for immune suppression, which could be targeted to enhance therapeutic efficacy [14]. The therapeutic implications are substantial; leveraging targeted IO against SIGLEC-15 in patients with low or absent PD-L1 expression might offer new avenues for treatment, potentially improving outcomes for a subset of patients who do not respond to conventional PD-1/PD-L1 therapies [15].

This study aims to evaluate the tumor-specific expression of SIGLEC-15 in gastric adenocarcinoma, its correlation with key clinicopathological parameters, and its impact on patient survival. In addition, this study assesses PD-L1 expression and investigates whether a potential relationship exists between SIGLEC-15 and PD-L1 in GC. A better understanding of the interplay between these immune markers may contribute to improved prognostic stratification and guide future therapeutic strategies involving immune modulation.

## 2. Results

The median age of the patient cohort was 68 years, with an interquartile range (IQR) of 59 to 75 years. Among all consecutive cases that met the inclusion criteria and were enrolled in this study, 73.6% were male. When age was dichotomized at the cohort median (68 years), PD-L1 positivity was 57.1% in <68 years versus (vs.) 52.9% in ≥68 years (*p* = 0.643), while SIGLEC-15 positivity was 44.4% vs. 41.4% (*p* = 0.7). Using the biologically motivated threshold of 60 years, the results were similar: PD-L1 was expressed in 52.9% of the patients <60 vs. 55.6% of those ≥60 (*p* = 0.843) and SIGLEC-15 in 50% vs. 40.4% (*p* = 0.4). In both approaches, no statistically significant differences were observed.

### 2.1. Associations Between SIGLEC-15 Expression and Clinicopathologic Features

For statistical analysis, cases were stratified into two categories based on the H-score value: SIGLEC-15 low H-score group (H-score < 110) and SIGLEC-15 high H-score group (H-score ≥ 110). The threshold of 110 was chosen as the median H-score within the study cohort, allowing a balanced and data-driven dichotomization of SIGLEC-15 expression for comparative analyses. There was no significant difference in Borrmann classification types III/IV (66.6% vs. 63.1%; *p* = 0.7), tumor size ≥ 5 cm (54.3% vs. 48.6%; *p* = 0.59), or in the rate of positive surgical margins (R1: 28.0% vs. 19.7%; *p* = 0.30). In addition, 24 patients (18%) had stage IV (pM1) disease, but without significant differences between the groups (pM1: 17.5% vs. 18.4%; *p* = 0.99). (Table 1).

However, several clinicopathologic parameters showed statistically significant associations with high SIGLEC-15 expression. Poorly differentiated tumors (G3) were more frequently observed in the high-expression group (61.4% vs. 46.0%; *p* = 0.03). Lymph node metastasis (pN1–3) was markedly more frequent in patients with high SIGLEC-15 expression (87.7% vs. 61.8%; *p* < 0.001). Lymphatic invasion (L1) was significantly more prevalent in the SIGLEC-15 high-expression group compared to the low-expression group (80.7% vs. 50.0%; *p* < 0.001). Similar findings were observed for venous invasion (V1: 54.3% vs. 30.2%; *p* = 0.007) and perineural invasion (Pn1: 49.1% vs. 25.0%; *p* = 0.005) (Table 1).

Although pT3–4 stages were more frequently observed in the SIGLEC-15 high group (91.2% vs. 81.5%), the difference did not reach statistical significance (*p* = 0.13) (Table 1).

In routine practice, first-line systemic therapy consisted of a fluoropyrimidine–platinum doublet per national standards. Anti-PD-1 was added only in the HER2-negative tumors meeting the Romanian PD-L1 thresholds (nivolumab for combined positive score (CPS) ≥ 5; pembrolizumab for CPS ≥ 10). Accordingly, 14/133 (10.5%) received anti-PD-1 plus chemotherapy (nivolumab 8/133; pembrolizumab 6/133), whereas 119/133 (89.5%) received systemic therapy without IO. Within the IO-treated subset (*n* = 14), SIGLEC-15 expression was high in 6/14 and low in 8/14 cases, with no significant differences.

### 2.2. Clinicopathological Characteristics Across SIGLEC-15 and PD-L1 Expression Subgroups

Based on PD-L1 CPS scoring, 60 patients (45.1%) were classified as CPS < 1 (PD-L1-negative), while 73 patients (54.9%) showed PD-L1 positivity (CPS ≥ 1). Among the PD-L1-positive group, 29 patients (21.8%) had CPS between 1–4, 18 patients (13.5%) had CPS between 5–9, and 26 patients (19.5%) had a CPS ≥ 10.

**Figure 1:**
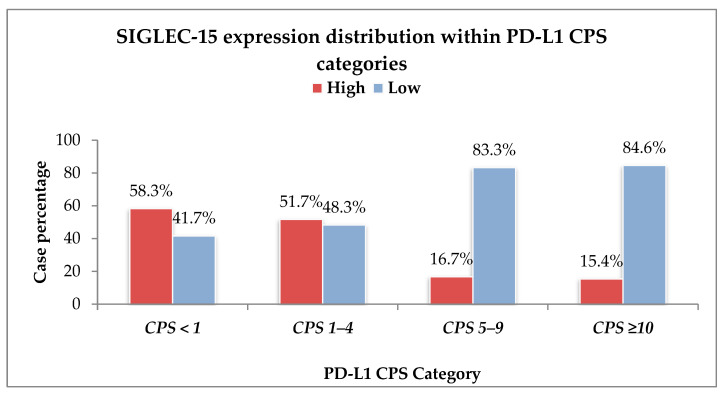
Bars indicate the proportion of cases with high (red) vs. low (blue) SIGLEC-15 expression within each PD-L1 CPS category. High SIGLEC-15 expression (H-score ≥ 110) was more frequently observed in PD-L1–negative cases (CPS < 1), while low SIGLEC-15 expression (H-score < 110) predominated in tumors with increasing PD-L1 expression (CPS ≥ 5 and CPS ≥ 10), suggesting an inverse relationship between the two immune markers.

A comparative assessment of SIGLEC-15 expression levels across PD-L1 CPS categories revealed distinct distributional trends (Figure 1).

High SIGLEC-15 expression (H-score ≥ 110) was more frequently observed in cases with CPS < 1 (58.3%) and CPS 1–4 (51.7%), while its expression markedly decreased in tumors with elevated CPS scores. Specifically, only 16.7% of cases in the CPS 5–9 subgroup and 15.4% of those with CPS ≥ 10 showed high SIGLEC-15 levels.

For further analysis, the patients were stratified into four subgroups based on their combined immune marker profiles:

(1) SIGLEC-15 low H-score (H-score < 110)/PD-L1 CPS < 1, *n* = 25 cases;
(2) SIGLEC-15 high H-score (H-score ≥ 110)/PD-L1 CPS < 1, *n* = 35 cases;
(3) SIGLEC-15 low H-score (H-score < 110)/PD-L1 CPS ≥ 1, *n* = 51 cases;
(4) SIGLEC-15 high H-score (H-score ≥ 110)/PD-L1 CPS ≥ 1, *n* = 22 cases.

This classification was used to investigate potential interactions between SIGLEC-15 and PD-L1 expression in relation to tumor aggressiveness.

Lymph node metastasis (pN1–3) was most frequently observed in both subgroups with high SIGLEC-15 expression, regardless of PD-L1 status. Specifically, 90.9% of patients in the SIGLEC-15 high H-score/PD-L1 CPS ≥ 1 subgroup and 86.7% in the SIGLEC-15 high H-score/PD-L1 CPS < 1 subgroup showed nodal involvement. In contrast, lower rates were noted in the SIGLEC-15 low H-score/PD-L1 CPS ≥ 1 (56.9%) and SIGLEC-15 low H-score/PD-L1 CPS < 1 (72%) groups. The differences across subgroups were statistically significant (*p* = 0.004). A similar tendency was observed for lymphatic invasion (L1), which affected 81.8% of cases with a high SIGLEC-15 H-score and positive PDL-1 status and 80% of those with high SIGLEC-15 expression and negative PD-L1 expression (CPS < 1), compared to the lower rates in the other subgroups (*p* = 0.003) (Table 2).

Venous invasion (V1) followed a similar pattern, being more common in patients with high SIGLEC-15 expression, regardless of PD-L1 status, reaching 54.5% and 54.3%, respectively (*p* = 0.030). Likewise, perineural invasion (Pn1) was found in half of the cases in the SIGLEC-15 high H-score/PD-L1-positive subgroup and 48.6% of the SIGLEC-15 high H-score/PD-L1-negative subgroup, while being notably less frequent in those with low SIGLEC-15 and PD-L1 positivity (19.6%) (*p* = 0.010) (Table 2).

No statistically significant differences were found between subgroups in terms of histologic grade, depth of invasion, distant metastasis, or surgical margin status (all *p* > 0.05) (Table 2).

### 2.3. Overall Survival Analysis According to SIGLEC-15 Expression

**Figure 2:**
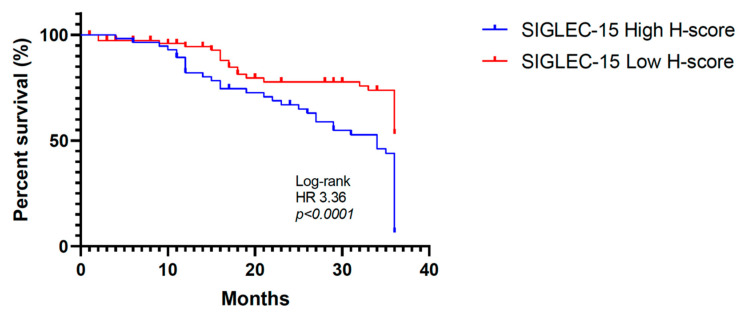
Impact of SIGLEC-15 expression on overall survival in GC. Kaplan–Meier curve showing significantly reduced overall survival in GC patients with high SIGLEC-15 expression (H-score ≥ 110) compared to those with low expression (H-score < 110).

The Kaplan–Meier survival analysis demonstrated that patients with high SIGLEC-15 expression (H-score ≥ 110) had significantly shorter overall survival compared to those with low expression (H-score < 110). At 36 months, the survival rate in the high-expression group was notably lower, with a hazard ratio (HR) of 3.36, a log-rank *p*-value < 0.0001, and 95% CI: 1.99–5.67 (Figure 2).

### 2.4. Survival Analysis Based on Combined SIGLEC-15 and PD-L1 Expression Patterns

**Figure 3:**
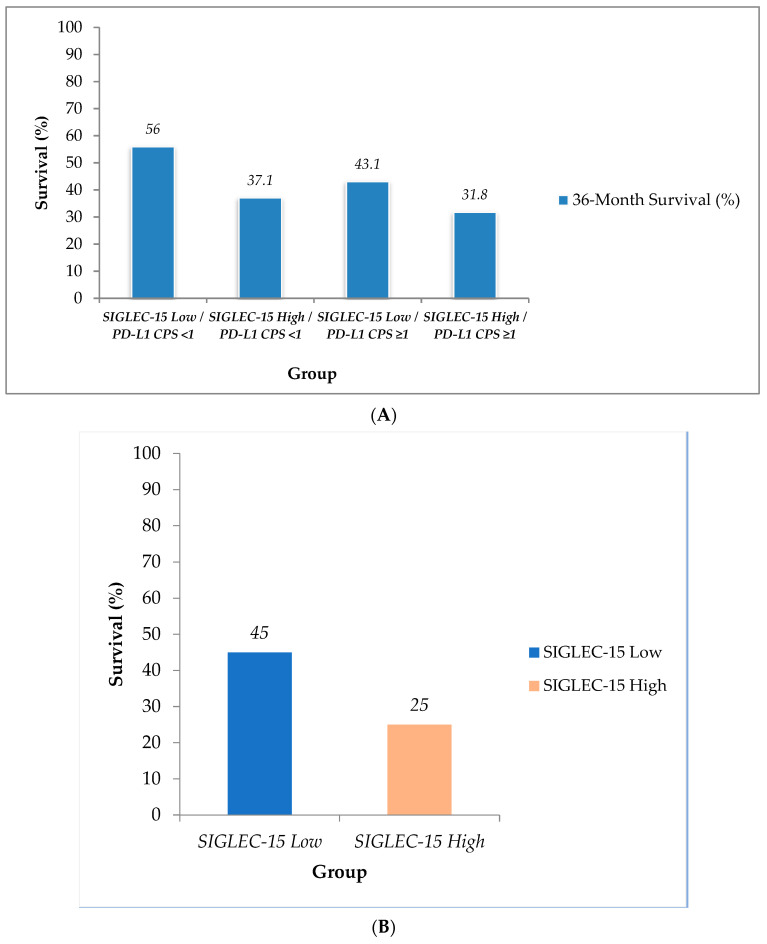
(**A**) Prognostic impact of combined SIGLEC-15/PD-L1 immunophenotype on 36-month survival. Patients were stratified by combined SIGLEC-15 expression (high vs. low) and PD-L1 CPS status (<1 vs. ≥1). SIGLEC-15 expression was classified as “Low” for H-score < 110 and “High” for H-score ≥ 110. (**B**) The 12-month overall survival (%) in the IO-treated subset (*n* = 14), stratified by SIGLEC-15 expression status (low vs. high). A 12-month time point was chosen due to the limited sample size and follow-up in this subgroup.

In the subgroup analysis based on combined SIGLEC-15 and PD-L1 status, the highest 36-month overall survival rate was seen in patients with SIGLEC-15 low H-score/PD-L1 CPS < 1 tumors (56%). This was followed by the SIGLEC-15 low H-score/PD-L1 CPS ≥ 1 group, with a 3-year survival rate of 43.1%. In contrast, the patients with tumors expressing high levels of SIGLEC-15 had worse outcomes, regardless of PD-L1 status. Specifically, the 36-month survival was 37.1% in the SIGLEC-15 high H-score/PD-L1 CPS < 1 group and 31.8% in the SIGLEC-15 high H-score/PD-L1 CPS ≥ 1 group (Figure 3A).

Among the 14 patients who received anti-PD-1 therapy, the 12-month survival rate was higher in the low SIGLEC-15 expression group (45%) compared with the high SIGLEC-15 expression group (25%) (Figure 3B).

**Figure 4:**
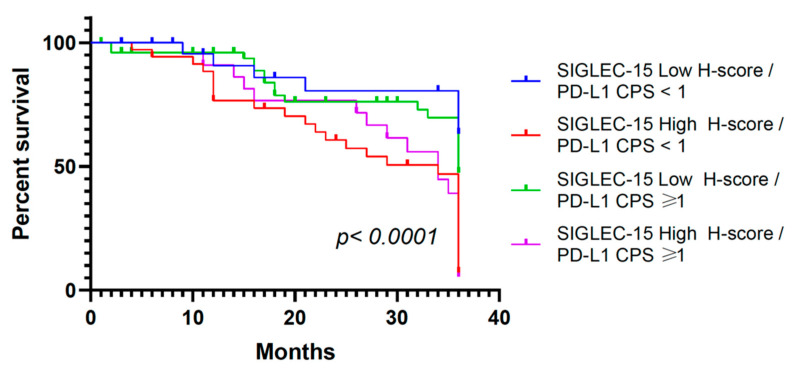
Impact on overall survival of SIGLEC-15 and PD-L1 expression in GC. Patients were stratified by combined SIGLEC-15 expression (high vs. low) and PD-L1 CPS status (<1 vs. ≥1). SIGLEC-15 expression was classified as “Low” for H-score < 110 and “High” for H-score ≥ 110.

Patients with concurrent high SIGLEC-15 expression (H-score ≥ 110) and positive PD-L1 status (CPS ≥ 1) had the poorest overall survival, with the survival curve declining more rapidly than in the other groups (*p* < 0.0001). In contrast, the most favorable prognosis was observed in the subgroup with low SIGLEC-15 expression and negative PD-L1 status (CPS < 1), which showed the highest overall survival rate throughout the 36 months. Intermediate outcomes were noted in the remaining two subgroups (SIGLEC-15 high/PD-L1 CPS < 1 and SIGLEC-15 low/PD-L1 CPS ≥ 1) (Figure 4).

## 3. Discussion

Since 2014–2015, the success of PD-1/PD-L1 inhibitors in various cancers has led researchers to seek other “checkpoint” molecules that tumors exploit. SIGLEC-15 was highlighted by Wang et al. in 2019 as a “next-generation” immune suppressor that is broadly upregulated across cancers and largely non-overlapping with PD-L1 [9,13,15]. In the last 5 years, multiple clinical studies have evaluated SIGLEC-15 expression in patient cohorts. For example, Ju Zhao et al. (2022) examined nasopharyngeal carcinoma and found a significant correlation between SIGLEC-15 and PD-L1 expression. Interestingly, both were favorable prognostic factors in that virus-associated cancer [16]. In anaplastic thyroid carcinoma and osteosarcoma, high SIGLEC-15 expression likewise predicted worse outcomes and was linked to the activation of pro-tumor pathways (STAT3/BCL-2) [9]. These studies reinforce that SIGLEC-15 expression often marks an immunosuppressive and aggressive tumor phenotype.

The literature on SIGLEC-15 in gastrointestinal (GI) malignancies has been relatively sparse until now. Apart from Quirino et al. (2021) in GC [13] and some bioinformatic analyses in colorectal cancer [17], few studies have addressed SIGLEC-15 expression clinically in GI tumors.

Our findings aim to fill the gap in the literature, demonstrating that elevated SIGLEC-15 expression in GC correlates with more aggressive pathological features and poorer survival outcomes. In our study, we used the Borrmann classification specifically to describe the macroscopic tumor growth patterns (types I–IV), as we aimed to capture the gross morphological features and their associations with invasive behavior and the TME. For the microscopic characterization, we reported the histologic differentiation grade (G1 = well, G2 = moderately, G3 = poorly differentiated) rather than Lauren’s classification, since our objective was to evaluate whether histologic grade, as a marker of tumor aggressiveness, correlates with the expression of SIGLEC-15 and PD-L1. However, we did not find associations between SIGLEC-15 expression and Borrmann types or advanced pT3-4 stages. Instead, in our cohort, SIGLEC-15 overexpression was significantly associated with indicators of tumor aggressiveness such as G3 grading, advanced tumor stage, and lymphovascular invasion. This aligns with emerging evidence across solid tumors that SIGLEC-15 upregulation is associated with unfavorable prognosis. A recent meta-analysis encompassing 13 studies (1376 patients) found that high SIGLEC-15 expression was linked to worse overall survival (pooled HR ~1.28) and associated with larger tumor size and advanced TNM stage [9]. Although the prognostic impact of SIGLEC-15 can vary by tumor type, the general trend in many cancers (including gastric, lung, and others) [9,18] is that SIGLEC-15-high tumors behave more aggressively. In the study published by Quirino et al. [13], SIGLEC-15 expression was evaluated in 71 GC patients and found in approximately 75 %, correlating with histologic grade and angiolymphatic invasion, but no significant association with survival outcomes was observed (OS *p* = 0.2692; DFS *p* = 0.6852). Given the small cohort size and short follow-up (28 months), the study may have lacked sufficient statistical power to detect a prognostic impact. In contrast, our study included a larger cohort (*n* = 133), with extended follow-up (36 months), enabling us to demonstrate that high SIGLEC-15 expression independently predicts worse overall survival, while also correlating with multiple aggressive pathological features.

In addition, our observation that SIGLEC-15-high tumors frequently presented with advanced stage mirrors findings in other GI malignancies. For example, in colorectal cancer, high SIGLEC-15 was associated with later stage and fewer tumor-infiltrating lymphocytes, indicative of immune evasion [17]. Mechanistically, SIGLEC-15 appears to facilitate an immunosuppressive tumor microenvironment (TME) that enables tumor growth and spread. SIGLEC-15 on tumor and myeloid cells can blunt anti-tumor immunity, thereby potentially allowing more aggressive tumor behavior [19,20]. This provides a plausible biological rationale for the worse outcomes observed in SIGLEC-15-high GC.

It is worth noting that the prognostic role of SIGLEC-15 is not uniformly negative across all settings. The impact may depend on the disease context and treatment background. For instance, in certain tumor settings, patients with SIGLEC-15-positive tumors had better response rates and improved survival compared to SIGLEC-15-negative cases. In these studies, SIGLEC-15 positivity (particularly when coupled with low PD-L1) predicted a favorable outcome, whereas PD-L1 positivity was associated with worse survival. The authors suggested that SIGLEC-15 expression might identify tumors with distinct therapeutic vulnerabilities and that SIGLEC-15 could serve as a biomarker for selecting IO strategies [16,18]. This contrasts with our findings (and most other reports), where SIGLEC-15 heralds a poor prognosis. The discrepancy highlights that SIGLEC-15’s prognostic significance can be context-dependent. Factors such as tumor histology, baseline immune infiltration, and treatments (whether patients received IO or chemoradiotherapy) may modulate the effect of SIGLEC-15.

Across tumor types, SIGLEC-15 IHC lacks a universally accepted scoring system. Studies have applied heterogeneous readouts, including H-score-based approaches (often dichotomized at study-specific thresholds), percentage-based tumor cell positivity cut-offs (e.g., ≥1%, ≥5%, ≥25%), semiquantitative immunoreactive schemes, or digital/multiplex image analysis. Some groups have evaluated tumor cells only, while others explicitly scored myeloid/macrophage compartments as well. For example, Quirino et al. applied an immunoreactive score system in GC [13], Chen et al. used a ≥5% membranous cut-off in pancreatic ductal adenocarcinoma [11], and Li et al. employed multiplex immunofluorescence with separate tumor cell and macrophage scoring in lung adenocarcinoma [21]. This methodological heterogeneity complicates cross-study comparisons and likely explains some of the variability in prognostic associations across different reports. Moreover, the meta-analysis of 13 studies (*n* = 1376) explicitly identified the “lack of a unified cutoff value” as a key source of heterogeneity [9].

Another key finding of our study is that SIGLEC-15 and PD-L1 show a generally inverse expression pattern in GC. This suggests that these two immune modulatory molecules may compensate for one another as alternate pathways of immune evasion. Our observations align with the growing body of evidence that SIGLEC-15 and PD-L1 expression are typically non-overlapping in many cancers [9,18]. Wang and colleagues first reported this phenomenon in lung cancer, identifying SIGLEC-15 as a novel immune checkpoint molecule that is mutually exclusive with PD-L1 in tumor tissues [15]. In that study, SIGLEC-15 was upregulated in many tumors that lacked PD-L1, and it was shown to suppress T cell responses independently of the PD-1/PD-L1 pathway [15]. The implication is that tumors that do not engage the PD-L1 axis may instead exploit SIGLEC-15 to escape immune surveillance [9,15]. Our findings in GC align with this paradigm. We found an inverse correlation between PD-L1 CPS and SIGLEC-15 levels, suggesting that these checkpoints are often utilized in an “either/or” manner in many cases.

Recent clinical and translational studies reinforce this inverse relationship. For instance, in non-small cell lung cancer (NSCLC), only a small minority of tumors co-express both PD-L1 and SIGLEC-15; one analysis reported the co-expression rate to be as low as ~3% [20]. Instead, PD-L1–negative NSCLC tumors frequently showed high SIGLEC-15 expression, especially on tumor-associated macrophages (TAMs) [20]. Those SIGLEC-15-high/PD-L1-low tumors were found to have an immune-excluded phenotype: SIGLEC-15-rich TAMs secreted high levels of immunosuppressive cytokines (e.g., interleukin (IL)-10) and low levels of stimulatory cytokines (e.g., IL-12), resulting in poor CD8+ T cell infiltration [20,22]. This underscores how a tumor may downregulate one checkpoint (PD-L1) and instead upregulate another (SIGLEC-15) to achieve a similar end, T-cell suppression.

It is important to clarify that “mutually exclusive” does not mean never co-expressed, but rather that the two markers typically do not overlap significantly at high levels. Our study emphasizes this nuance: while most cases favor one pathway (either PD-L1 or SIGLEC-15), a subset did co-express both pathways at certain levels. GC patients whose tumors co-expressed high SIGLEC-15 and had PD-L1 CPS ≥ 1 experienced the worst survival of any subgroup. They also showed the most aggressive pathological features (deep invasion, nodal metastases, etc.). In our cohort, this dual-positive subgroup is a particularly high-risk category. The combined negative effect on survival suggests that simultaneous engagement of both immunosuppressive pathways, myeloid-driven and T-cell/adaptive, creates a highly suppressive tumor environment, allowing the tumor to evade immune control more effectively than by either mechanism alone [23,24]. For example, the overexpression of PD-L1 alongside other non-redundant checkpoints such as V-domain Ig suppressor of T cell activation or lymphocyte-activation gene 3 has been linked to significantly suppressed T-cell activity and resistance to IO [15,25,26]. In the case of PD-L1 and SIGLEC-15, one acts at the T-cell–antigen-presenting cell/tumor cell interface (through PD-1/PD-L1 inhibitory signaling), while the other can influence myeloid cells and their interaction with T cells. Co-expression could therefore shut down anti-tumor immunity from multiple angles at once [27].

The management of resectable GC in our country largely follows the principles outlined in the European Society for Medical Oncology (ESMO) and National Comprehensive Cancer Network (NCCN) guidelines, which recommend radical surgery combined with perioperative chemotherapy [28,29]. Similarly, for advanced or unresectable disease, immune checkpoint inhibitors such as nivolumab or pembrolizumab are increasingly incorporated into treatment regimens for selected patients, in line with European Medicines Agency (EMA)-approved indications [30]. In our center, treatment decisions are made within a multidisciplinary tumor board, which systematically reviews each case and decides the management according to the tumor stage, biological profile, and patient-specific factors. This integrative approach ensures optimal alignment with international standards while allowing individualized strategies, particularly regarding the choice between curative surgery and palliative procedures, the sequencing of systemic therapy, and the potential inclusion of IO. According to our results, patients with tumors that are high in dual checkpoints might benefit the least from standard therapies and may need innovative or combination IO, supporting the relevance of such individualized therapies. For instance, if the GC expresses PD-L1 (making it eligible for anti-PD-1 therapy) but also has high SIGLEC-15 expression, one could expect primary or early-acquired resistance to the PD-1/PD-L1 blockade due to the presence of an alternative suppressive pathway. This idea is supported by murine cancer models, where blocking SIGLEC-15 has been shown to restore T-cell function and work synergistically with the PD-1 blockade in resistant tumors [9,15,25,26]. In the same study, it was observed that SIGLEC-15 can facilitate immune escape in PD-L1-knockout tumors, and combining anti-SIGLEC-15 with anti-PD-1 resulted in better tumor control in mouse models compared to using either alone [15]. Although clinical data are still limited, it is reasonable to suggest that patients who are dual-positive might benefit from therapies targeting both checkpoints at the same time [31].

A first-in-class humanized anti-SIGLEC-15 antibody, NC318, has been in Phase I testing for advanced solid tumors (NCT03665285), and preliminary reports have shown signs of clinical benefits in patients with SIGLEC-15-positive tumors, including partial responses in IO-resistant lung and ovarian cancers [32]. While no trials specific to GC have been reported yet, our findings provide a strong rationale to include GC patients in such studies, particularly those whose tumors are PD-L1-negative or refractory to PD-1 blockade.

### Strengths, Limitations, and Future Perspectives

Our study focuses on the histopathological characteristics of gastric adenocarcinoma and the expression of SIGLEC-15, examining its relationship with established markers of tumor aggressiveness and its prognostic impact on overall survival. Both the analysis and the discussion are primarily based on histologic parameters, aiming to provide a clearer understanding of how SIGLEC-15 expression integrates with GC. Given that original research studies investigating SIGLEC-15 expression in GC are still scarce, we believe our findings provide valuable insights, demonstrating robust correlations with histopathologic features and offering a transparent, reproducible methodology that supports future research and improved risk stratification.

In our cohort, high SIGLEC-15 was linked with adverse pathology and shorter overall survival, and its distribution was partly non-overlapping with PD-L1; the dual-positive group had the poorest outcomes. We believe these observations could be useful in advanced disease by considering SIGLEC-15 alongside PD-L1 in exploratory biomarker panels. Broadly, a PD-L1-positive/SIGLEC-15-low profile may align with current PD-1-based approaches per guidelines, whereas SIGLEC-15-high tumors (especially PD-L1-low or dual-positive cases) might be candidates for consideration in clinical trials testing myeloid/SIGLEC-15-directed combinations with chemotherapy ± PD-1. We view these points as hypothesis-generating, intended to inform biomarker panels and trial design, and will allow closer follow-up strategies for these high-risk cases.

A limitation of the present study lies in the use of the H-score method for evaluating SIGLEC-15 expression, which focused exclusively on tumor cells and did not account for positive staining in stromal or immune cells, such as macrophages. Although SIGLEC-15 positivity was occasionally observed in these non-tumor compartments, such expression was not quantitatively assessed. In contrast, PD-L1 expression is scored using the CPS, which integrates both tumor and immune cell staining, offering a more complex immunological landscape. The absence of a standardized scoring system for SIGLEC-15 across studies further contributes to variability and limits the comparability of results in the current literature. As such, our findings should be interpreted within this context.

As a future direction, we aim to develop or incorporate a scoring system that also quantifies SIGLEC-15 expression in the stromal and immune microenvironment, particularly in macrophages and lymphocytes. To support this effort, future studies will also consider multiplex IHC approaches to better characterize the specific cellular sources of SIGLEC-15 positivity in the TME. At this stage, however, we focused our analysis on tumor cell expression, given the lack of robust data on SIGLEC-15 in GC and the need to establish baseline patterns before expanding into more complex spatial assessments.

## 4. Material and Methods

### 4.1. Study Population and Clinicopathologic Data

This was a retrospective, cross-sectional study designed to characterize baseline PD-L1 and SIGLEC-15 expression in treatment-naïve GC specimens, which enrolled 133 patients diagnosed with gastric adenocarcinoma between 2020 and 2021 at the Pathology Department of the County Clinical Hospital Târgu Mureș, Romania. The inclusion criteria were as follows: (1) histologically confirmed diagnosis of gastric adenocarcinoma; (2) availability of formalin-fixed, paraffin-embedded (FFPE) tissue blocks; (3) no history of neoadjuvant therapy before surgical resection; and (4) accessible complete clinicopathological data.

All patients provided informed consent before participation. This study was conducted following the principles of the Declaration of Helsinki and received approval from the Ethics Committee of the County Clinical Hospital Târgu Mureș, Romania (approval number 13106/09.09.2024).

Clinical and pathological data were obtained through a comprehensive review of pathology reports and surgical records. The analyzed variables included patient age, sex, tumor size, histologic grade (well, moderately, or poorly differentiated), Borrmann classification (grouped as type I–II vs. type III–IV), depth of invasion (pT category), lymph node status (pN), distant metastasis (pM), lymphatic invasion (L), venous invasion (V), perineural invasion (Pn), and resection margin status (R0/R1). Histopathological diagnosis and classification of gastric adenocarcinoma were performed following the 5th edition of the WHO Classification of Tumors: Digestive System Tumors [33]. Tumor staging was determined according to the 8th edition of the American Joint Committee on Cancer (AJCC) Staging Manual [34].

Data regarding survival status and follow-up duration were retrieved from institutional medical records. Out of the 133 included cases, 129 patients had complete 36-month follow-up data available, while 4 patients were censored due to incomplete survival information.

### 4.2. Immunohistochemistry (IHC)

Immunohistochemical staining was performed on all formalin-fixed, FFPE tumor samples to assess the expression of PD-L1 and SIGLEC-15.

**Figure 5:**
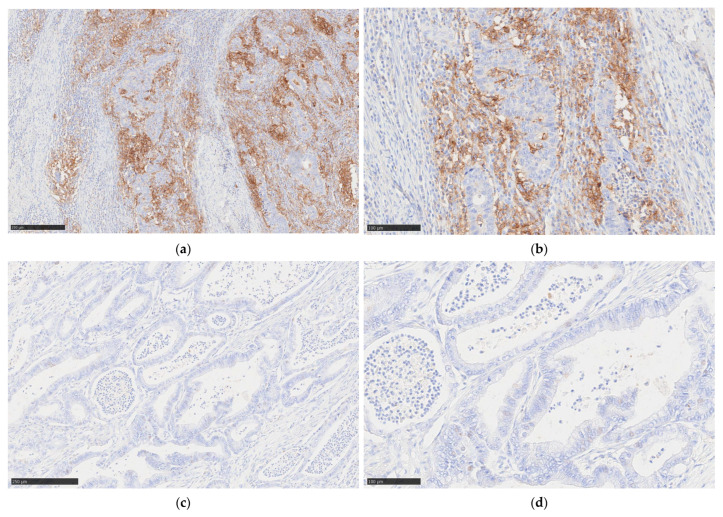
Representative immunohistochemical images of PD-L1 expression in GC cases with different CPS levels. (**a**) Low-magnification view of a GC case with positive PD-L1 expression (CPS ≥ 1), showing membranous staining in tumor cells and associated immune cells; (**b**) high-magnification view of the same PD-L1-positive case (CPS ≥ 1), highlighting distinct PD-L1 staining in both tumor and immune components; (**c**) low-magnification view of a GC case with PD-L1 negativity (CPS < 1), showing absence of PD-L1 staining; (**d**) high-magnification view of the same CPS < 1 case, confirming lack of PD-L1 expression in tumor and immune cells.

PD-L1 expression was evaluated using the 22C3 clone (Agilent Technologies, Santa Clara, CA, USA), following the manufacturer’s standardized protocol. PD-L1 expression was assessed using the CPS, calculated as the number of PD-L1-stained tumor cells, lymphocytes, and macrophages divided by the total number of viable tumor cells, multiplied by 100. This scoring system follows international guidelines for GC using the Agilent 22C3 PharmDx assay. A CPS < 1 was considered negative, while a CPS ≥ 1 indicated PD-L1 positivity (Figure 5).

In routine care, PD-L1 was not uniformly available at treatment decision-making; therefore, for this study we retrospectively performed PD-L1 IHC on archived FFPE blocks for all cases lacking a prior clinical result (78.9%). These study-specific assessments did not influence the patients’ management.

**Figure 6:**
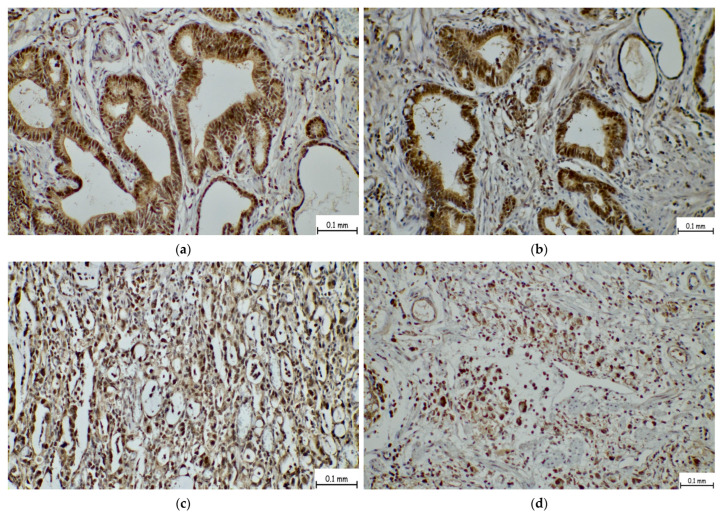
Representative immunohistochemical staining of SIGLEC-15 with a high H-score in gastric adenocarcinoma. (**a**,**b**) Moderately differentiated gastric adenocarcinoma showing strong and diffuse SIGLEC-15 expression, corresponding to a high H-score. (**c**,**d**) Poorly differentiated gastric adenocarcinoma with high SIGLEC-15 expression; image (**d**) additionally demonstrates areas with signet-ring cell features exhibiting similar high H-score-based staining intensity.

**Figure 7:**
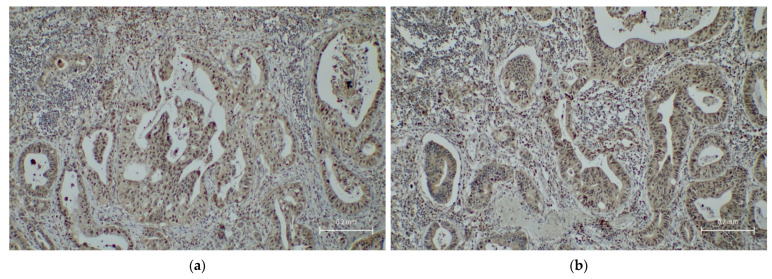

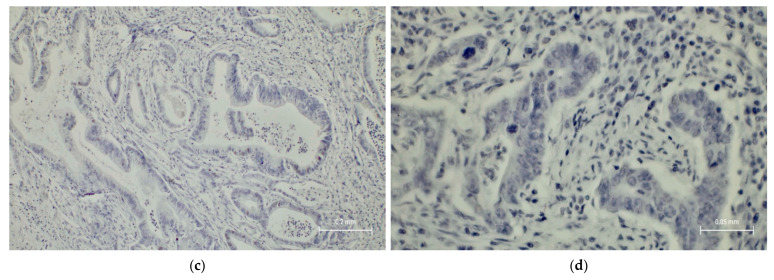
Representative images of SIGLEC-15 with a low H-score in gastric adenocarcinoma. (**a**,**b**) Moderately differentiated gastric adenocarcinoma with weak and focal staining for SIGLEC-15, corresponding to a low H-score pattern; (**c**,**d**) negative control for SIGLEC-15 in gastric adenocarcinoma.

SIGLEC-15 expression was evaluated using a rabbit polyclonal antibody targeting the N-terminal region of SIGLEC-15 (ab174732, Abcam, Cambridge, UK), applied at a 1:300 dilution, following the manufacturer’s recommended protocol for FFPE specimens. The level of expression was semi-quantitatively evaluated by two independent pathologists using the H-score system, which incorporates both the staining intensity and the percentage of positive tumor cells. The H-score was calculated as follows:

H-score = [1 × (% of weakly stained cells) + 2 × (% of moderately stained cells) + 3 × (% of strongly stained cells)], resulting in a total score ranging from 0 to 300 (Figure 6 and Figure 7).

All slides were independently evaluated manually by two pathologists using a Zeiss Axio Lab A1 light microscope (Carl Zeiss Microscopy GmbH, Jena, Germany). In cases of discrepancy, a consensus score was reached through joint evaluation.

### 4.3. Statistical Analysis

All statistical analyses were performed using GraphPad Prism, version 10.4.1 (GraphPad Software, Boston, MA, USA). Continuous variables are reported as median values with IQRs, while categorical variables are summarized as absolute frequencies and percentages. Comparisons between categorical variables, including associations between PD-L1 expression (CPS categories) and SIGLEC-15 expression groups, were assessed using chi-square or Fisher’s exact test. The relationship between continuous CPS scores and SIGLEC-15 expression (high vs. low) was evaluated using the Mann–Whitney U test. A two-tailed *p*-value < 0.05 was considered statistically significant. Survival analysis was conducted using the Kaplan–Meier method, with group differences assessed by the log-rank test. Survival time was calculated from the date of diagnosis to the last follow-up or death. Bar charts and survival plots (Figure 1 and Figure 3) were created in Microsoft Excel (Microsoft Office 365, Microsoft Corporation, Redmond, WA, USA).

## 5. Conclusions

Our study highlights the clinical significance of SIGLEC-15 expression in GC. High SIGLEC-15 expression was significantly associated with adverse pathological features, including lymphatic, venous, and perineural invasion, as well as reduced overall survival, compared to cases with low expression. Moreover, when stratified by combined SIGLEC-15 and PD-L1 CPS status, patients co-expressing both markers (SIGLEC-15 high/PD-L1 CPS ≥ 1) exhibited the poorest survival outcomes, while those negative for both had the most favorable prognosis. These findings suggest that SIGLEC-15 not only reflects tumor aggressiveness but also adds prognostic value when evaluated alongside PD-L1. Dual assessment may help refine patient stratification and support the rationale for developing combined immune checkpoint strategies in GC.

## Figures and Tables

**Table 1:** Comparison of clinicopathological parameters between high and low SIGLEC-15 expression groups in GC.

Parameter	SIGLEC-15 High H-Score Group *n* = 57		SIGLEC-15 Low H-Score Group *n* = 76		Fisher’s *p*-Value *	OR	95% CI
	*n*	%	*n*	%			
Male sex	42	73.6	56	73.6	0.99	1.00	0.46–2.18
Age ≥68 years	29	50.8	41	53.9	0.72	0.88	0.44–1.76
Borrmann classification III and IV	38	66.6	48	63.1	0.71	1.17	0.57–2.40
Tumor size ≥5 cm	31	54.3	37	48.6	0.59	1.26	0.63–2.50
Grading G3 (poorly differentiated)	35	61.4	35	46.0	0.03	2.12	1.05–4.28
Tumor invasion depth pT3-4	52	91.2	62	81.5	0.13	2.35	0.79–6.95
Lymph node metastasis pN1-3	50	87.7	47	61.8	<0.001	4.41	1.76–11.02
Distant metastasis status pM1	10	17.5	14	18.4	0.99	0.94	0.38–2.31
Lymphatic invasion (L1)	46	80.7	38	50.0	<0.001	4.18	1.89–9.28
Venous invasion (V1)	31	54.3	23	30.2	0.007	2.75	1.34–5.62
Perineural invasion (Pn1)	28	49.1	19	25.0	0.005	2.90	1.39–6.04
Positive surgical margins (R1)	16	28.0	15	19.7	0.30	1.59	0.71–3.56
Systemic therapy							
Anti-PD-1 + chemotherapy vs. chemotherapy without IO	6	10.5	8	10.5	0.99	1.00	0.33–3.06

* Obtained from Fisher’s exact test; OR: odds ratio; CI: 95% confidence interval; IO: immunotherapy.

**Table 2:** Comparison of clinicopathologic characteristics across combined SIGLEC-15 and PD-L1 expression subgroups in GC.

Parameter	SIGLEC-15 Low H-Score/PD-L1 CPS < 1 (*n* = 25)	SIGLEC-15 High H-Score/PD-L1 CPS < 1 (*n* = 35)	SIGLEC-15 Low H-Score/PD-L1 CPS ≥ 1 (*n* = 51)	SIGLEC-15 High H-Score/PD-L1 CPS ≥ 1 (*n* = 22)	*p*-Value *
Grading G3 Poorly differentiated	12 (48%)	20 (57.1%)	23 (45.1%)	15 (68.2%)	0.28
Tumor invasion depth pT3-4	22 (88%)	31 (88.6%)	40 (78.4%)	21 (95.4%)	0.08
Lymph node metastasis pN1-3	18 (72%)	30 (85.7%)	29 (56.9%)	20 (90.9%)	0.004
Distant metastasis status pM1	4 (16%)	5 (14.3%)	10 (19.6%)	5 (22.7%)	0.84
Lymphatic invasion (L1)	14 (56%)	28 (80%)	24 (47.1%)	18 (81.8%)	0.003
Venous invasion (V1)	6 (24%)	19 (54.3%)	17 (33.3%)	12 (54.5%)	0.03
Perineural invasion (Pn1)	9 (36%)	17 (48.6%)	10 (19.6%)	11 (50%)	0.01
Positive surgical margins (R1)	4 (16%)	11 (31.4%)	11 (21.6%)	5 (22.7%)	0.54

* Obtained from the chi-square test; CPS = combined positive score.

## Data Availability

The datasets used and analyzed during the current study are available from the corresponding author upon request.

## Abbreviations

AJCC	American Joint Committee on Cancer
CPS	Combined positive score
EMA	European Medicines Agency
ESMO	European Society for Medical Oncology
FFPE	Formalin-fixed: paraffin-embedded
GC	Gastric cancer
GI	Gastrointestinal
IHC	Immunohistochemistry
IQR	Interquartile ranges
IL	Interleukin
IO	Immunotherapy
NCCN	National Comprehensive Cancer Network
NSCLC	Non-small cell lung cancer
PD-L1	Programmed death-ligand 1
SIGLEC-15	Sialic acid-binding immunoglobulin-like lectin 15
TAMs	Tumor-associated macrophages
